# miRNA and mRNA Expression Profiles Associated with Lymph Node Metastasis and Prognosis in Penile Carcinoma

**DOI:** 10.3390/ijms23137103

**Published:** 2022-06-26

**Authors:** Claudio B. Murta, Tatiane K. Furuya, Alexis G. M. Carrasco, Miyuki Uno, Laura Sichero, Luisa L. Villa, Sheila F. Faraj, Rafael F. Coelho, Giuliano B. Guglielmetti, Mauricio D. Cordeiro, Katia R. M. Leite, William C. Nahas, Roger Chammas, José Pontes

**Affiliations:** 1 Urology Department, Instituto do Cancer do Estado de Sao Paulo (ICESP), Hospital das Clinicas da Faculdade de Medicina da Universidade de Sao Paulo (HCFMUSP), Sao Paulo CEP 01246-000, SP, Brazil; rafael.coelho@hc.fm.usp.br (R.F.C.); giuliano.betoni@hc.fm.usp.br (G.B.G.); mauricio.cordeiro@hc.fm.usp.br (M.D.C.); katiaramos@usp.br (K.R.M.L.); wnahas@uol.com.br (W.C.N.); docjpjr@uol.com.br (J.P.J.); 2 Center for Translational Research in Oncology (LIM24), Instituto do Cancer do Estado de Sao Paulo (ICESP), Hospital das Clinicas da Faculdade de Medicina da Universidade de Sao Paulo (HCFMUSP), Sao Paulo CEP 01246-000, SP, Brazil; agmurilloc@usp.br (A.G.M.C.); miyuki.uno@hc.fm.usp.br (M.U.); laura.sichero@hc.fm.usp.br (L.S.); l.villa@hc.fm.usp.br (L.L.V.); rchammas@usp.br (R.C.); 3 Pathology Department, Instituto do Cancer do Estado de Sao Paulo (ICESP), Hospital das Clinicas da Faculdade de Medicina da Universidade de Sao Paulo (HCFMUSP), Sao Paulo CEP 01246-000, SP, Brazil; sheilafaraj@yahoo.com.br

**Keywords:** disease progression, metastasis, microRNA and mRNA expression levels, penile cancer, prognosis, survival analysis, tumor biomarkers

## Abstract

Penile cancer (PeC) is a rare disease, and no prognostic biomarkers have been adopted in clinical practice yet. The objective of the present study was to identify differentially expressed miRNAs (DEmiRs) and genes (DEGs) as potential biomarkers for lymph node metastasis and other prognostic factors in PeC. Tumor samples were prospectively obtained from 24 patients with squamous cell carcinoma of the penis. miRNA microarray analysis was performed comparing tumors from patients with inguinal lymph node metastatic and localized disease, and the results were validated by qRT-PCR. Eighty-three gene expression levels were also compared between groups through qRT-PCR. Moreover, DEmiRs and DEGs expression levels were correlated with clinicopathological variables, cancer-specific (CSS), and overall survival (OS). TAC software, TM4 MeV 4.9 software, SPSS v.25.0, and R software v.4.0.2 were used for statistical analyses. We identified 21 DEmiRs in microarray analysis, and seven were selected for validation. miR-744-5p and miR-421 were overexpressed in tissue samples of metastatic patients, and high expression of miR-421 was also associated with lower OS. We found seven DEGs (*CCND1*, *EGFR*, *ENTPD5*, *HOXA10*, *IGF1R*, *MYC*, and *SNAI2*) related to metastatic disease. A significant association was found between increased *MMP1* expression and tumor size, grade, pathological T stage, and perineural invasion. Other genes were also associated with clinicopathological variables, CSS and OS. Finally, we found changes in mRNA–miRNA regulation that contribute to understanding the mechanisms involved in tumor progression. Therefore, we identified miRNA and mRNA expression profiles as potential biomarkers associated with lymph node metastasis and prognosis in PeC, in addition to disruption in mRNA–miRNA regulation during disease progression.

## 1. Introduction

Penile cancer (PeC) is a rare disease, with 36,000 new cases estimated worldwide for 2020 [1]. Its treatment is mainly based on partial or total penectomy [2], even among patients with early disease stages [3]. After treatment of the primary tumor, almost half of the patients are submitted to bilateral inguinal lymphadenectomy, which accounts for complications in 55% of cases [4,5]. Inguinal metastasis is the most important clinical prognostic factor for cancer-specific survival (CSS) [5], and it is present in almost 50% of patients [6]. Unfortunately, clinicians have used only clinical and pathological features to stratify patients who might benefit from lymphadenectomy, which may end in unnecessary procedures, thus emphasizing the importance of identifying biomarkers associated with metastatic progression [2]. 

Some molecular biomarkers were described as prognostic factors, but none have been applied to clinical practice yet [2]. The discovery of new biomarkers could help to support the implementation of an affordable clinical test that could reduce the overtreatment by lymphadenectomy, mainly in middle and low-income countries, where PeC is more prevalent [7].

Recently, epigenetic alterations have emerged to provide insights into the carcinogenesis of PeC, with a special focus on microRNAs (miRNAs) [8,9,10,11,12,13,14]. However, few studies have focused on the prognostic value of miRNA expression in PeC. In 2016, Hartz et al. were the first group to describe miRNAs associated with metastatic potential in PeC [10].

This study aimed to identify miRNA and mRNA expression profiles as potential biomarkers for lymph node metastasis and other prognostic factors in a prospective PeC cohort. We also investigated the deregulation of mRNA–miRNA pairs associated with disease progression. 

## 2. Results

### 2.1. Description of the Sample

Among 41 cases of PeC diagnosed at ICESP between 2015 and 2018, 24 were included in this study. The exclusion criteria for patients are detailed in Table S3. 

Table 1 depicts clinicopathological data for all 24 PeC patients and separately by samples used in microarray analysis (*n* = 11) and in an independent (*n* = 13) cohort. The mean age at diagnosis ± Standard Deviation (SD) was 61.8 ± 16.1 years old, and the median follow-up was 39.8 months. Partial penectomy was performed in 17 patients (70.8%), and all specimens were classified as the usual histological subtype of squamous cell carcinoma (SCC). Eleven patients had inguinal metastasis confirmed by pathology, and one was classified as cN3. At the analysis time, ten patients (41.7%) had died from any cause, and seven (29.2%) due to PeC. HPV DNA was detected in tumors from eight patients (33.3%) as described in our previous study [14]. 

### 2.2. miRNA and mRNA Expression as Prognostic Markers for Lymph Node Metastasis and Association with Clinicopathological Variables

Microarray analysis detected 21 DEmiRs when comparing tumors obtained from patients with metastatic (*n* = 6) and localized (*n* = 5) disease. Table S4 shows the complete list of DEmiRs with their respective fold changes (FC) and p-values. The hierarchical clustering analysis and the reasons for choosing specific DEmiRs for validation are depicted in Figure S1A. The selected DEmiRs were not validated using the independent group of samples (Table 2; Figure S1B). However, miR-744-5p and miR-421 expression levels showed association with lymph node metastasis in the whole cohort of PeC patients (*n* = 24) (Table 2). Both miRNAs also presented high area under the curve (AUC) values in the Receiver Operating Characteristic (ROC) curve, showing their ability to distinguish patients with metastasis (Figure S2). 

We identified seven upregulated DEGs (*CCND1*, *EGFR*, *ENTPD5*, *HOXA10*, *IGF1R*, *MYC*, and *SNAI2*) in the tumors of patients with metastatic (Table 3) in relation to localized disease. ROC curves of these DEGs showed high AUC values and good accuracy in discriminating patients with lymph node metastasis (Figure S2). We also associated the mRNA levels with other clinicopathological variables (Table 3). Overexpression of *MMP1* levels was significantly associated with tumor size >5cm, grade III, presence of perineural invasion, and a worse T stage and was shown to be a good predictor for all of these clinicopathological variables according to the high AUC values (Figure S3). *CCND1* and *EGFR* were upregulated in tumors with grade III, showing good accuracy according to AUC values (Figure S4). Figure S4 demonstrates other transcripts as good predictors for other clinicopathological variables.

### 2.3. mRNA–miRNA Ratios Proposed as Prognosis Biomarkers for PeC 

**Figure 1:**
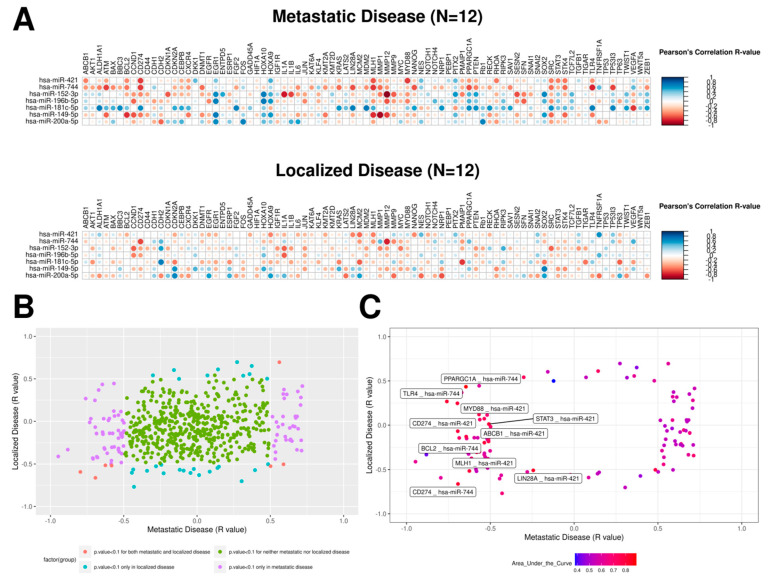
Pair-wise correlations between expression levels of seven miRNAs and 83 genes in tumors of patients with metastatic and localized disease in the total sample of 24 PeC patients. (**A**) Pearson correlation coefficients between the expression levels of miRNAs and mRNAs showed different profiles across metastatic and localized diseases. The size and color of each dot represent the Pearson correlation R-values for each combination of mRNA–miRNA pairs; (**B**) Scatter plot for all mRNA–miRNA pair-wise ratios in metastatic and localized tissues. Each dot was colored according to the *p*-value for each tissue; (**C**) Scatter plot for mRNA–miRNA pairs with significant correlations in at least one tissue. Dots are colored according to their Area Under the Curve (AUC) values for discrimination of metastatic (*n =* 12) and localized tumor (*n =* 12) samples.

We plotted all Pearson correlation values obtained between mRNA and miRNA expression levels for tumors of patients with metastatic (*n* = 12) and localized disease (*n* = 12) (Figure 1A), and we observed a different pattern comparing both panels. To improve the accuracy of the mRNA–miRNA pair selection, we colored all mRNA–miRNA pairs according to their *p*-values in both tissues (Figure 1B) and selected pairs with *p*-values lower than 0.1 in at least one of the groups (117 out of 581). Next, we performed a ROC analysis and found ten mRNA–miRNA pairs with good accuracy for discriminating tumors of patients with metastatic or localized disease (AUC > 0.8; Figure 1C; Figure S5A). Among these pairs, we identified two miRNAs (miR-744 and miR-421) and nine mRNAs (*ABCB1*, *BCL2*, *CD274*, *LIN28A*, *MLH1*, *MYD88*, *PPARGC1A*, *STAT3*, and *TLR4*). We observed that *CD274* appeared twice in this analysis (related to both miRNAs). Seven out of ten pairs (miR-421 and *ABCB1*, *CD274*, *MYD88*, and *STAT3* and miR-744 and *BCL2*, *PPARGC1A* and *TLR4*) presented disruption in their regulation patterns, changing from no correlation or positive correlation in tumors of patients with the localized disease to a negative correlation in metastatic disease (Figure S5).

### 2.4. Association of miRNA and mRNA Levels with OS and CSS

**Figure 2:**
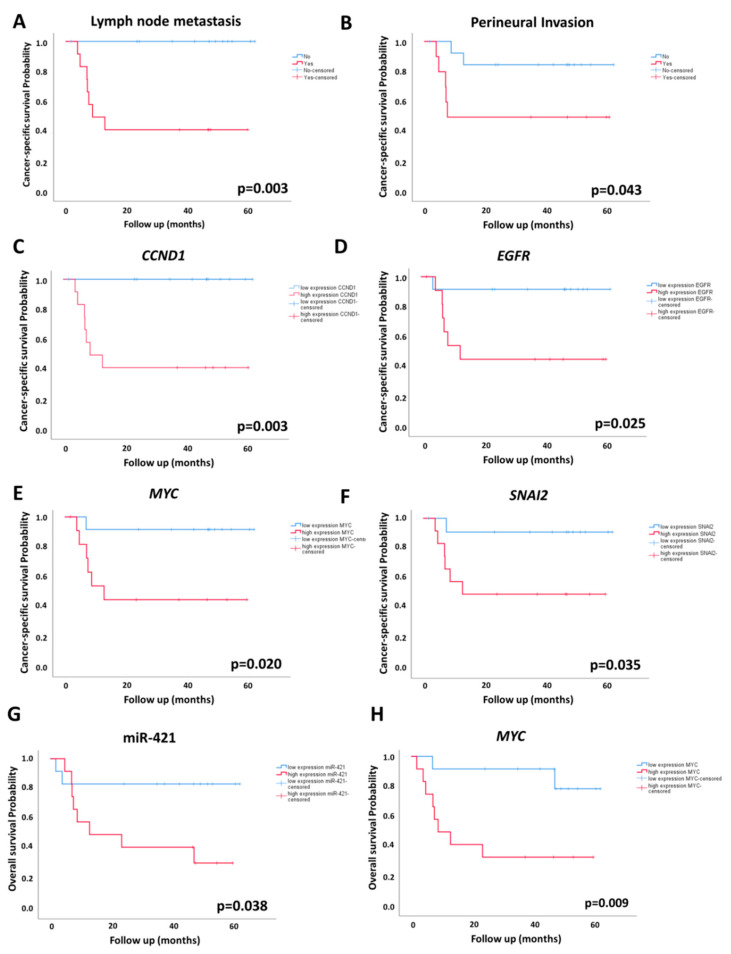
Cancer-specific survival (CSS) (**A**–**F**) and overall survival (OS) (**G**,**H**) analyses of PeC patients according to the clinicopathological variables and miRNA/gene expression patterns. Kaplan–Meier curves show that the presence of lymph node metastasis (**A**) and perineural invasion (**B**) were associated with worse CSS; High expression (defined as values above the -dCT median expression) of *CCND1* (**C**), *EGFR* (**D**), *MYC* (**E**), and *SNAI2* (**F**) were associated with shorter CSS; miR-421 (**G**) and *MYC* (**H**) were associated with shorter OS.

The means of OS and CSS were 40.9 and 45.3 months, respectively. The maximum follow-up time was 62 months. Clinicopathological characteristics and their association with OS and CSS are summarized in Table S5. The presence of lymph node metastasis and perineural invasion was associated with worse CSS (Figure 2A,B). No clinicopathological variables were associated with OS (*p* > 0.05).

Table 4 describes all significant associations between miRNA and mRNA levels with CSS and OS. Regarding miRNA expression, we observed that overexpression of miR-421 was associated with shorter OS (Figure 2G; Table 4).

Considering the DEGs that were upregulated in lymph node metastasis, we found that overexpression of *CCND1*, *EGFR*, *MYC*, and *SNAI2* genes were associated with shorter CSS (Figure 2; Table 4). *MYC* overexpression was also associated with OS (Figure 2; Table 4). Other genes related to survival are presented in Table 4, Figures S6 and S7.

## 3. Discussion

We performed a prospective translational study aiming to identify molecular signatures based on miRNA and gene expression levels associated with the prognosis of PeC and thereby discovering new prognostic and target therapy biomarkers. We found that overexpression of miR-421, miR-744-5p, and a panel of seven genes were associated with metastatic disease in PeC patients. Despite recent advances, no biomarkers have been established to guide the decision about performing inguinal lymphadenectomy [2], and no systemic treatment so far has achieved good results for patients with metastatic disease [2]. Therefore, new biomarkers related to metastatic progression and targets for therapy are urgently needed.

For the first time, we demonstrated that miR-421 and miR-744-5p were upregulated in metastatic PeC. Overexpression of miR-421 was also associated with lymph node metastasis in non-small-cell lung cancer (NSCLC) [15] and gastric cancer [16]. miR-744-5p was found to be upregulated in NSCLC patients with lymph node metastasis and poor prognosis [17] and elevated in plasma levels of pancreatic cancer with lymph node metastasis [18].

Few studies have investigated the role of miRNA as a prognostic factor of PeC. To the best of our knowledge, only four studies have described miRNAs associated with metastasis in PeC [10,11,12,13]. In 2016, Hartz et al. described three miRNAs (miR-1, miR-101, and miR-204) and, in 2017, Kuasne et al. identified other three miRNAs (miR-17-5p, miR-106a-5p, and miR-512-3p) downregulated in tumors of PeC patients with lymph node metastasis [10,11]. More recently, Pinho et al. (2020) and Ayoubian et al. (2021) described that the upregulation of miR-223-3p [12] and downregulation of miR-137 and miR-328-3p [13], respectively, were also associated with lymph node metastasis.

We also detected seven upregulated genes in PeC patients with metastatic disease tumors. *CCND1*, *EGFR,* and *MYC* were shown to be altered in PeC, with DNA amplification or copy number variations being associated with a poor prognosis [19,20]. Furthermore, EGFR-targeted therapies were clinically tested in PeC patients [21]. The *SNAI2* gene encodes a family member of zinc finger transcriptional factors involved in the epithelial–mesenchymal transition process by inhibiting the transcription of the E-cadherin gene [22]. Although *SNAI2* mRNA expression level is associated with lymph node metastasis in tongue SCC [23], this is the first time it was associated with lymph node metastasis in PeC. These genes showed high accuracy in discriminating patients with metastatic disease, potentially avoiding an unnecessary inguinal lymphadenectomy, which occurs in up to 70% of cases [5].

Although *MMP1* is associated with several worse prognostic factors in our study, no association with lymph node metastasis was found as described in the study of Kuasne et al. in PeC [11].

In addition to the analysis that showed the alterations of miRNA and mRNA profiles in PeC progression separately, we also investigated mRNA–miRNA pairs that were deregulated during tumor progression. We highlighted four mRNA–miRNA pairs that include genes related to the immune response: miR-421/*CD274*, miR-421/*MYD88*, miR-744-5p/*CD274,* and miR-744-5p/*TLR4*. The *CD274* gene encodes the PD-L1 protein, which binds to its receptor in the T Cell (PD1), shutting it off and reducing the immunological response [24]. PD-L1 expression was shown to be present in up to 32% of PeC and is associated with shorter CSS [25]. The other two genes (*MYD88* and *TLR4*) have been implicated in the innate immune response; part of this response is due to the activation of *TLR4* by *MYD88*, which releases pro-inflammatory cytokines [26]. The *MYD88* has never been related to carcinogenesis or prognosis in PeC. However, the low expression of the TLR4 protein was observed in patients with HPV infection [27].

Regarding survival, we found that miR-421 overexpression was associated with shorter OS, similar to the reports in NSCLC [15] and gastric cancer [28]. This is the first description of a single miRNA related to overall survival in PeC. In the literature, the low expression of all three miRNAs (miR-1, miR-101, and miR-204) was previously associated with shorter CSS in PeC [10].

Moreover, overexpression of *CCND1*, *EGFR*, *MYC*, and *SNAI2* genes were also associated with shorter CSS and overexpression of *MYC* with shorter OS. Those genes have already been associated with shorter CSS or OS in PeC or other types of SCC [19,23]. We also showed that *GADD45A* overexpression was associated with shorter CSS and OS, with the highest HR values (20.89 and 6.43, respectively). *GADD45A* has never been investigated in PeC and is related to DNA damage response [29]. It acts as an oncogene or a tumor suppressor gene depending on its response to oncogenic stimuli [30].

One limitation of the study was the small sample size due to the rarity of PeC. This could explain why some DEmiRs detected by microarray analysis were not validated by qRT-PCR. It is also important to mention that, because ICESP is a tertiary referral center, we had a small proportion of patients with pT1 disease. Nevertheless, the presence of lymph node metastasis was balanced in our sample. Further studies with larger PeC cohorts are required to confirm our findings and functionally validate the detected mRNAs–miRNAs interactions.

Overall, this study has several strengths. We prospectively followed a PeC cohort for a long period and uniformly treated patients according to guidelines, making clinical endpoints stronger. Another relevant factor was using a microarray technique to identify DEmiRs instead of studying a small panel chosen based on a literature search. Using an innovative analysis, we demonstrated changes in the regulation of mRNA–miRNA pairs during disease progression, enlarging the horizon for future research in PeC. 

## 4. Materials and Methods

### 4.1. Study Population

From July 2015 to January 2018, patients with pathologically confirmed SCC of the penis were prospectively enrolled in our study. They were all newly diagnosed localized or locally advanced cases referred for surgical treatment with curative intent. The exclusion criteria were the presence of other malignancies at diagnosis, previous treatment for PeC, previous pelvic radiotherapy, palliative surgery without curative intent, patients who underwent treatment for the primary tumor in another institution, Human Immunodeficiency Virus (HIV), Hepatitis B or C infections. Fresh frozen PeC tumoral tissues were collected and stored in the Academic Biobank for Research on Cancer at the University of Sao Paulo (USP), Instituto do Cancer do Estado de Sao Paulo (ICESP), Sao Paulo, Brazil.

This study was approved by the Local Ethics Committee (Protocol 1.016.980) and carried out under the terms of the Helsinki Declaration. The study and Biobank’s written informed consent and epidemiological questionnaire were obtained from all participants.

Medical history and physical examination data were obtained during hospital admission. Pathological tumor data such as tumor size, histology, grade, staging, lymphovascular and perineural invasions, margins, and lymph node metastasis, were collected from the patients’ anatomopathological reports and medical records. Metastatic disease was defined as metastasis to inguinal or pelvic lymph nodes, either clinically or pathologically confirmed, or distant metastasis.

The disease was staged by computed tomography imaging of the chest, abdomen, and pelvis. Patients underwent partial or total penectomy according to the extent of the disease and were prospectively followed from the time of enrollment until April 2021 according to the European Association of Urology (EAU) Guidelines on PeC [2]. Patients with T1 stage, well or moderately differentiated tumor, and no signs of inguinal metastases (by physical examination or computed tomography scan) were only followed up on, whereas those patients with clinical metastases, poorly differentiated tumor, or ≥T2 stage underwent bilateral radical inguinal lymphadenectomy [2].

### 4.2. Total RNA Extraction 

PeC frozen tissues (30 mg) were used for total RNA extraction using the miRVana^TM^ miRNA Isolation kit (Thermo Fisher Scientific, MA, USA), according to the manufacturer’s instructions. RNA concentration and purity were determined, and only samples with an A260/A280 ratio between 1.8 and 2.1 and an RNA Integrity Number above seven were included.

### 4.3. miRNA Expression Profiling

The miRNA expression profile was investigated in samples from patients with metastatic (n = 6) or localized disease (*n* = 5) using the GeneChip miRNA 4.0 Array (Thermo Fisher Scientific) following the manufacturer’s protocol. The samples were hybridized on the array, stained in the Affymetrix Fluidics Station 450, and scanned with the Affymetrix GeneChip Scanner 3000 7G. Expression Console software (Thermo Fisher Scientific) was used for the quality assessment of microarray data. Transcriptome Analysis Console (TAC) software version 4.0.2 (Thermo Fisher Scientific) was used for background correction, normalization, and summarization of raw data (CEL files) by Robust Multichip Average plus Detection Above Background (RMA + DABG). We used the Linear Models for Microarrays (LIMMA) test with at least 1.5-fold changes (FC > 1.5) and *p* < 0.05 to identify differentially expressed miRNAs (DEmiRs) between samples from patients with metastatic (*n* = 6) or localized disease (*n* = 5). The TAC software constructed hierarchical clustering graphs with the DEmiRs selected for further validation. Microarray datasets are available on the Gene Expression Omnibus database (GSE172095).

### 4.4. Validation of DEmiRs by Quantitative Reverse Transcription Polymerase Chain Reaction (qRT-PCR)

The DEmiRs identified from microarray analysis were selected for validation by qRT-PCR in an independent set of PeC samples (*n* = 13) and whole cohort (*n* = 24). Total RNA was reverse transcribed to cDNA using the TaqMan^®^ Advanced miRNA cDNA Synthesis Kit (Thermo Fisher Scientific). The miRNA levels were quantified using Taqman^®^ Advanced miRNA Assays (Table S1) (Thermo Fisher Scientific). miR-103a-3p and miR-423-5p were chosen as endogenous controls. Samples were run in triplicate, and FC was calculated using the comparative CT method (2^−ΔΔCT^) [31]. We compared miRNA expression levels in tumors of patients with metastatic and localized disease using Student’s *t*-test and *p* < 0.05 in TM4 MultiExperiment Viewer (MeV) 4.9 software. Boxplots were constructed using SPSS v.25.0.

### 4.5. mRNA Expression Profiling

We explored DEGs in all samples of patients with metastatic (*n* = 12) or localized disease (*n* = 12) using a panel containing 90 genes (83 of interest and seven endogenous; Table S2). Gene expression levels were evaluated by qRT-PCR using the nanofluidic platform Biomark™ HD System Real-Time PCR (Fluidigm, South San Francisco, CA, USA), according to the manufacturer’s instructions. We chose a panel that included genes targeted by the identified DEmiRs from the microarray analysis, genes related to carcinogenesis, epithelial–mesenchymal transition process, DNA damage response, cell cycle, and genes previously described as deregulated in PeC and other SCC. D3 Assay Design software (Fluidigm) was used for designing the Delta Gene™ assays containing forward and reverse primers.

A total of 45 ng RNA was used for cDNA synthesis (Fluidigm), followed by 10 cycles of pre-amplification reaction, which was performed using a pool of 90 Delta Gene™ assays (Fluidigm) representing all investigated genes at a final concentration of 500 nM each. Pre-amplified cDNAs were treated with Exonuclease I (New England BioLabs, Ipswich, MA, USA) and diluted 1:10.

Gene expression levels were quantified by qRT-PCR using the EvaGreen^®^ dye method (Bio-Rad Laboratories, Hercules, CA, USA) and were prepared according to the manufacturer’s instructions. Delta Gene™ Assays (final concentration of 5 µM) and solutions were pipetted into the 96.96 Dynamic Array Integrated Fluidic Circuit (IFC) according to the manufacturer’s recommended pipetting map and then placed on the Juno IFC controller (Fluidigm) to load samples and assays into the 96.96 Dynamic Array IFC. Next, qRT-PCRs were conducted using the nanofluidic platform Biomark™ HD System Real-Time PCR (Fluidigm) according to the established protocol. Results were extracted from the Biomark Data Collection version 4.5.1 software and were analyzed using the Fluidigm Real-Time PCR Analysis version 4.3.1 software (Fluidigm). Obtained values were plotted individually for each gene, and only samples with CT values lower than 24 were considered for analysis.

NormFinder, a Microsoft^®^ Excel add-in [32], was used to assess the stability of expression levels of the seven endogenous control genes (*ACTB*, *B2M*, *GAPDH*, *GUSB*, *HPRT1*, *RPLP0*, and *TFRC*) used as candidates to normalize qRT-PCR data. Based on each gene’s intra- and inter-group variations, this program could automatically determine the most stably expressed candidate reference genes and gene pairs in a sample. *ACTB* and *RPL0* were the most stable endogenous genes in our samples. FC was calculated using the comparative CT method (2^−ΔΔCT^) [31]. The TM4 MeV 4.9 software was used to detect DEGs in the comparison of tumors of patients with metastatic (*n* = 12) or localized disease (*n* = 12) in the total sample of PeC patients by using Student’s t-test and *p* < 0.05.

### 4.6. Human Papillomavirus (HPV) Detection 

After paraffin removal using xylene, PeC samples were digested with proteinase K/SDS 0.1% for 24 h. DNA was obtained by phenol:chloroform extraction, and samples were diluted to 50 ng/µL. The quality of the DNA was determined by amplifying a 110 bp fragment of the human β-globin gene with PCO3/PCO4 primers, followed by analysis on an 8% acrylamide gel electrophoresis [33]. The Inno-LiPA HPV Genotyping kit (Innogenetics, Gent, Belgium) was used for HPV DNA detection and genotyping as previously described [34].

### 4.7. Statistical Analyses

To analyze differences between samples used in the microarray analysis and the independent or expanded validation, we used Chi-square or Fisher’s Exact, Mann–Whitney tests, and *p* < 0.05 as significant in SPSS version 25.0. The association of miRNAs and DEGs expression levels with clinicopathological variables was accessed using Student’s t-test in TM4 MeV 4.9 software, considering *p* < 0.05 as significant.

The ROC curves were constructed using SPSS version 25.0. The AUC values were calculated to analyze the accuracy of each DEmiR and DEG for distinguishing tumors of patients with metastatic and localized diseases with specificity and sensitivity. The same analysis was performed for all the other clinicopathological variables of the study. For the mRNA–miRNA pairs identified in the correlation analyses, ROC curves analysis was performed to evaluate the prognostic testability of these pairs (using the ratio values of -delta CT mRNA and -delta CT miRNA).

Pearson’s correlation coefficients for tumors of patients with metastatic or localized disease were performed in the R software v. 4.0.2 (“Hmisc” package) using expression data for seven miRNAs and 83 genes. The “tidyr” package was loaded to select mRNA–miRNA pairs with significant *p*-values (≤0.1) for at least one of the tissues. We estimated these selected pairs’ mRNA/miRNA ratio using the “PRROC” package to calculate AUC values. Subsequently, the “corrplot” and “ggplot2” packages were used to plot correlation values. Finally, we used the “ggrepel” package to tag mRNA–miRNA pairs with AUC values higher than 0.8.

Cancer-specific (CSS) and overall survival (OS) analyses were performed using SPSS v.25.0 and *p* < 0.05 value as significant. We used the Kaplan–Meier method, and the statistical differences were analyzed using the log-rank test. We performed a Cox regression model to estimate the Hazard Ratio (HR) and 95% confidence intervals (CI) values. High and low miRNA and transcript expression levels in tumor samples were defined by the median of -dCT values.

## 5. Conclusions

We detected two miRNAs and seven genes overexpressed in tumors of patients with metastatic disease in a prospective cohort of PeC patients. We also demonstrated that these miRNAs and genes showed good accuracy in identifying patients with metastasis and that they are possible potential biomarkers to be used in PeC. Additionally, we found that the upregulation of miR-421 was associated with shorter OS, and the expression of 19 genes was associated with other clinicopathological variables, ten with CSS and seven with OS in PeC. Finally, we detected changes in mRNA–miRNA regulation that could help to explain the tumor progression process.

## Figures and Tables

**Table 1:** Clinicopathological characteristics of PeC patients separated by each set of samples.

Patients	Total Sample *n* (%)	Microarray Sample *n* (%)	Validation Sample *n* (%)	*p*
**Number of patients**	24	11	13	
**Age at surgery–Mean (SD) years old**	61.8 (16.1)	61.6 (14.2)	61.9 (18.2)	0.931
**Follow up–Median (range) months**	39.8 (2–68)	47.5 (8–62)	34.9 (2–61)	
**cT**				0.378
cT1	3 (12.5)	2 (18.2)	1 (7.7)	
cT2	13 (5.2)	4 (36.4)	9 (69.2)	
cT3	8 (33.3)	5 (45.4)	3 (23.1)	
**cN**				0.327
cN0	9 (37.5)	5 (45.4)	4 (30.8)	
cN1	8 (33.3)	3 (27.3)	5 (38.4)	
cN2	4 (16.7)	3 (27.3)	1 (7.7)	
cN3	3 (12.5)	0 (0)	3 (23.1)	
**Penectomy**				0.476
Partial	17 (70.8)	7 (63.6)	10 (76.9)	
Total	7 (29.2)	4 (36.4)	3 (23.1)	
**Grade**				0.854
I	3 (12.5)	2 (18.2)	1 (7.7)	
II	13 (54.2)	6 (54.5)	7 (53.9)	
III	8 (33.3)	3 (27.3)	5 (38.4)	
**T Stage**				0.246
pT1	4 (16.6)	3 (27.2)	1 (7.7)	
pT2	13 (54.2)	4 (36.4)	9 (69.2)	
pT3	7 (29.2)	4 (36.4)	3 (23.1)	
**Inguinal Lymphadenectomy**	16 (66.7)	7 (63.6)	9 (69.2)	0.772
**Pelvic Lymphadenectomy**	4 (16.7)	2 (18.1)	2 (15.4)	1.000
**Lymph node metastasis**	12 (50.0)	6 (54.5)	6 (46.2)	0.682
**HPV DNA detection**	8 (33.3)	3 (27.3)	5 (38.4)	0.562
**Tumor Size (>5 cm)**	8 (33.3)	5 (45.5)	3 (23.1)	0.247
**Lymphovascular invasion**	6 (25.0)	3 (27.3)	3 (23.1)	0.813
**Perineural invasion**	10 (41.7)	4 (36.4)	6 (46.2)	0.628
**Group Risk (EAU)**				0.381
Low	1 (4.2)	1 (9.1)	0 (0)	
Intermediate	3 (12.5)	2 (18.2)	1 (7.7)	
High	20 (83.3)	8 (72.7)	12 (92.3)	

PeC: penile cancer; *n*: number of patients; SD: standard deviation; HPV: human papillomavirus; EAU: European Association of Urology; *p* values refer to the comparison between validation (*n* = 13) and microarray samples (*n* = 11). No differences were found between groups (*p* > 0.05).

**Table 2:** List of seven differentially expressed miRNAs (DEmiRs) identified by microarray analysis (*n* = 11) when comparing tumor samples of patients with metastatic in relation to localized disease and chosen for validation in the independent (*n* = 13) and in the total cohort of patients (*n* = 24) by qRT-PCR.

Regulation	DEmiR	*Microarray*		qRT-PCR Independent Cohort		qRT-PCR Total Cohort	
		FC	*p*	FC	*p*	FC	*p*
Up	miR-421	2.89	<0.001 *	2.12	0.051	2.21	0.005 *
	miR-149-5p	6.30	0.002 *	0.77	0.367	1.92	0.375
	miR-744-5p	1.65	0.001 *	1.36	0.190	1.61	0.003 *
	miR-200a-5p	2.10	0.047 *	0.90	0.600	1.00	0.686
	miR-152-3p	2.60	0.033 *	1.15	0.928	1.48	0.243
	miR-196b-5p	1.66	0.025 *	1.00	0.504	1.33	0.550
Down	miR-181c-5p	0.63	0.031 *	1.68	0.126	1.17	0.851

DEmiRs: differentially expressed miRNAs; *n*: number of individuals; PeC: penile cancer; FC: fold change; SD: standard deviation; qRT-PCR: quantitative Reverse Transcription Polymerase Chain Reaction; * *p* < 0.05.

**Table 3:** Significant associations between gene expression levels and clinicopathological variables in the total sample of PeC patients (*n* = 24).

Clinicopathological Variables	mRNA	FC	*p*
Lymph node metastasis (yes vs. no)	*IGF1R*	1.44	0.020
	*MYC*	1.55	0.028
	*CCND1*	1.63	0.034
	*SNAI2*	1.82	0.010
	*ENTPD5*	2.04	0.011
	*HOXA10*	2.24	0.014
	*EGFR*	3.69	0.006
Perineural Invasion (yes vs. no)	*MMP9*	0.28	0.044
	*KLF4*	0.47	0.040
	*TP53I3*	1.68	0.034
	*MMP1*	5.43	0.007
Lymphovascular Invasion (yes vs. no)	*IL1A*	3.29	0.027
pT stage (T2 + T3 vs. T1)	*ABCB1*	0.39	0.020
	*MMP1*	7.44	0.018
Histological Grade (III vs. I + II)	*RIPK3*	0.60	0.037
	*SRC*	1.68	0.005
	*CCND1*	1.92	0.006
	*MMP1*	3.91	0.045
	*EGFR*	5.27	0.001
HPV infection (presence vs. absence)	*SNAI2*	0.54	0.014
	*BAX*	0.72	0.030
Tumor Size (> 5 cm vs. ≤ 5 cm)	*LIN28A*	0.36	0.047
	*NANOG*	0.40	0.042
	*TP53*	0.50	0.014
	*MMP1*	3.98	0.042

PeC: penile cancer; *n*: number of individuals; FC: Fold Change; HPV: human papillomavirus; Student *t*-test; *p* < 0.05.

**Table 4:** Cancer-specific and overall survival when comparing high and low expression levels of miRNAs and transcripts.

Survival	miRNA or mRNA	*p* Log-Rank	HR (95% CI)	*p*
Cancer-specific survival ^#^	*CCND1*	0.003 *	115.14 (0.71–18810)	0.068
	*CDH1*	0.014 *	0.254 (0.03–2.25)	0.219
	*CDKN1A*	0.039 *	0.615 (0.07–5.74)	0.669
	*EGFR*	0.025 *	2.40 (0.28–20.65)	0.425
	*GADD45A*	0.018 *	20.89 (1.47–297.0)	0.025 *
	*KLF4*	0.031 *	0.36 (0.04–3.08)	0.352
	*MYC*	0.020 *	2.92 (0.34–24.9)	0.327
	*SNAI2*	0.035 *	3.98 (0.46–34.63)	0.212
	*SRC*	0.027 *	13.92 (0.95–204.1)	0.055
	*TP63*	0.046 *	0.20 (0.02–1.80)	0.153
Overall survival	miR-421	0.038 *	4.50 (0.95–21.33)	0.058
	*CDH1*	0.001 *	0.07 (0.01–0.56)	0.012 *
	*FGF2*	0.009 *	6.23 (1.30–29.84)	0.022 *
	*GADD45A*	0.008 *	6.43 (1.34–30.84)	0.020 *
	*IL6*	0.045 *	3.68 (0.94–14.36)	0.061
	*MYC*	0.009 *	6.31 (1.31–30.29)	0.021 *
	*NES*	0.015 *	5.56 (1.17–26.37)	0.031 *
	*NRP1*	0.013 *	5.78 (1.22–27.36)	0.027 *

High and low miRNA and transcript expression levels were defined as values above the median of expression levels (-dCT values); HR: hazard ratio; ^#^ adjusted by lymph node metastasis and perineural invasion; CI: confidence interval; * *p* < 0.05.

## Data Availability

Microarray datasets are available on the Gene Expression Omnibus (GEO) database (accession number GSE172095).

## Supplementary Materials

The following supporting information can be downloaded at: https://www.mdpi.com/article/10.3390/ijms23137103/s1.
