# Supplementary material for: miRNA and mRNA Expression Profiles Associated with Lymph Node Metastasis and Prognosis in Penile Carcinoma
Source: Int J Mol Sci. 2022 Jun 26;23(13):7103. doi: 10.3390/ijms23137103 (PMC9266734; doi:10.3390/ijms23137103)
Supplement: Supplementary file 1 [file ijms-23-07103-s001.zip › ijms-1768611-supplementary.pdf]

Supplementary Material

Table S1. Taqman® Advanced miRNA Assays of the differentially expressed miRNAs (DEmiRs) selected for validation by qRT-PCR.

Type	DEmiR	TaqMan® Advanced miRNA Assays
DEmiRs selected for validation	miR-181c-5p	477934_mir
	miR-744-5p	478200_mir
	miR-196b-5p	478585_mir
	miR-200a-5p	478752_mir
	miR-152-3p	477921_mir
	miR-421	478088_mir
	miR-149-5p	477917_mir
Endogenous controls	miR-103a-3p	478253_mir
	miR-423-5p	478090_mir

DEmiR: differentially expressed miRNA; qRT-PCR: quantitative Reverse Transcription Polymerase Chain Reaction.

Table S2. Complete list of the genes included in the panel (83 genes of interest and seven endogenous controls) used to evaluate transcript expression levels in the total sample of penile cancer patients (*n*=24).

Gene symbol	Gene Name	Classification
ABCB1	*ATP binding cassette subfamily B member 1*	Gene of interest
ACTB	*Actin Beta*	Endogenous control
AKT1	*AKT serine/threonine kinase 1*	Gene of interest
ALDH1A1	*aldehyde dehydrogenase 1 family member A1*	Gene of interest
ATM	*ATM serine/threonine kinase*	Gene of interest
B2M	*Beta-2-Microglobulin*	Endogenous control
BAX	*BCL2 associated X, apoptosis regulator*	Gene of interest
BBC3	*BCL2 binding component 3*	Gene of interest
BCL2	*BCL2, apoptosis regulator*	Gene of interest
CCND1	*cyclin D1*	Gene of interest
CD274	*CD274 molecule*	Gene of interest
CD44	*CD44 molecule (Indian blood group)*	Gene of interest
CDH1	*cadherin 1*	Gene of interest
CDH2	*cadherin 2*	Gene of interest
CDKN1A	*cyclin dependent kinase inhibitor 1A*	Gene of interest
CDKN2A	*cyclin dependent kinase inhibitor 2A*	Gene of interest
CEBPB	*CCAAT/enhancer binding protein beta*	Gene of interest
CXCR4	*C-X-C motif chemokine receptor 4*	Gene of interest
DKK1	*dickkopf WNT signaling pathway inhibitor 1*	Gene of interest
DNMT1	*DNA methyltransferase 1*	Gene of interest
EGFR	*epidermal growth factor receptor*	Gene of interest
EGR1	*early growth response 1*	Gene of interest
ENTPD5	*ectonucleoside triphosphate diphosphohydrolase 5*	Gene of interest
ESRP1	*epithelial splicing regulatory protein 1*	Gene of interest
FGF2	*fibroblast growth factor 2*	Gene of interest
FOS	*Fos proto-oncogene, AP-1 transcription factor subunit*	Gene of interest
GADD45A	*growth arrest and DNA damage inducible alpha*	Gene of interest
GAPDH	*Glyceraldehyde-3-Phosphate Dehydrogenase*	Endogenous control
GUSB	*Glucuronidase Beta*	Endogenous control
HIF1A	*hypoxia inducible factor 1 alpha subunit*	Gene of interest
HOXA10	*homeobox A10*	Gene of interest
HOXA9	*homeobox A9*	Gene of interest
HPRT1	*Hypoxanthine Phosphoribosyltransferase 1*	Endogenous control
IGF1R	*insulin-like growth factor 1 receptor*	Gene of interest

IL1A	*interleukin 1 alpha*	Gene of interest
IL1B	*interleukin 1 beta*	Gene of interest
IL6	*interleukin 6*	Gene of interest
JUN	*Jun proto-oncogene, AP-1 transcription factor subunit*	Gene of interest
KAT6A	*lysine acetyltransferase 6A*	Gene of interest
KLF4	*Kruppel like factor 4*	Gene of interest
KMT2A	*lysine methyltransferase 2A*	Gene of interest
KMT2D	*lysine methyltransferase 2D*	Gene of interest
KRAS	*KRAS proto-oncogene, GTPase*	Gene of interest
LATS2	*large tumor suppressor kinase 2*	Gene of interest
LIN28A	*lin-28 homolog A*	Gene of interest
MCM2	*minichromosome maintenance complex component 2*	Gene of interest
MDM2	*MDM2 proto-oncogene*	Gene of interest
MLH1	*mutL homolog 1*	Gene of interest
MMP1	*matrix metallopeptidase 1*	Gene of interest
MMP12	*matrix metallopeptidase 12*	Gene of interest
MMP9	*matrix metallopeptidase 9*	Gene of interest
MYC	*v-myc avian myelocytomatosis viral oncogene homolog*	Gene of interest
MYD88	*myeloid differentiation primary response 88*	Gene of interest
NANOG	*Nanog homeobox*	Gene of interest
NES	*nestin*	Gene of interest
NOTCH1	*notch 1*	Gene of interest
NOTCH4	*notch 4*	Gene of interest
NRP1	*neuropilin 1*	Gene of interest
PEBP1	*phosphatidylethanolamine binding protein 1*	Gene of interest
PITX2	*paired like homeodomain 2*	Gene of interest
PMAIP1	*phorbol-12-myristate-13-acetate-induced protein 1*	Gene of interest
PPARGC1A	*PPARG coactivator 1 alpha*	Gene of interest
PTEN	*phosphatase and tensin homolog*	Gene of interest
Rb1	*RB transcriptional corepressor 1*	Gene of interest
RECK	*reversion inducing cysteine rich protein with kazal motifs*	Gene of interest
RHOA	*ras homolog family member A*	Gene of interest
RIPK3	*receptor interacting serine/threonine kinase 3*	Gene of interest
RPLP0	*Ribosomal Protein Lateral Stalk Subunit P0*	Endogenous control
SAV1	*salvador family WW domain containing protein 1*	Gene of interest
SESN2	*sestrin 2*	Gene of interest
SFN	*stratifin*	Gene of interest
SNAI1	*snail family transcriptional repressor 1*	Gene of interest
SNAI2	*snail family transcriptional repressor 2*	Gene of interest

SOX2	*SRY-box 2*	Gene of interest
SRC	*SRC proto-oncogene, non-receptor tyrosine kinase*	Gene of interest
STAT3	*signal transducer and activator of transcription 3*	Gene of interest
STK4	*serine/threonine kinase 4*	Gene of interest
TCF7L2	*transcription factor 7like 2*	Gene of interest
TFRC	*Transferrin Receptor*	Endogenous control
TGFB1	*transforming growth factor beta 1*	Gene of interest
TIGAR	*TP53 induced glycolysis regulatory phosphatase*	Gene of interest
TLR4	*toll-like receptor 4*	Gene of interest
TNFRSF1A	*TNF receptor superfamily member 1A*	Gene of interest
TP53	*tumor protein p53*	Gene of interest
TP53I3	*tumor protein p53 inducible protein 3*	Gene of interest
TP63	*tumor protein p63*	Gene of interest
TWIST1	*twist family bHLH transcription factor 1*	Gene of interest
VEGFA	*vascular endothelial growth factor A*	Gene of interest
WNT5a	*Wnt family member 5A*	Gene of interest
ZEB1	*zinc finger E-box binding homeobox 1*	Gene of interest

Table S3. Total number of PeC patients included in the study and reasons for the exclusion of 17 patients.

**Patients of the study**	***n***
Number of PeC patients at ICESP	41
Exclusion criteria of patients	17
Patient refused to sign the informed consent	2
Patient refused penectomy	1
Death before treatment	3
Advanced disease	2
Previous treatment for PeC	1
HIV or HCV infection	2
Penectomy performed in another Hospital	2
Insufficient material	3
Lost to follow-up	1
Total number of included patients	24

n: number of patients; ICESP: Instituto do Câncer do Estado de São Paulo; PeC: penile carcinoma; HIV: human immunodeficiency virus; HCV: hepatitis C virus.

Table S4. Complete list of the 21 DEmiRs identified by microarray analysis when comparing tumors of patients with metastatic (*n*=6) in relation to those with localized (*n*=5) disease.

Regulation	DEmiR	Fold Change	p
Downregulation	miR-7641	0.38	0.0274
	miR-4443	0.50	0.0462
	miR-1293	0.50	0.0478
	miR-181c-5p	0.63	0.0314
	miR-5100	0.63	0.0010
Upregulation	miR-1296-5p	1.59	0.0067
	miR-877-5p	1.60	0.0364
	miR-4446-3p	1.62	0.0110
	miR-1307-3p	1.63	0.0013
	miR-744-5p	1.65	0.0010
	miR-196b-5p	1.66	0.0245
	miR-4428	1.70	0.0262
	miR-1910-5p	1.70	0.0335
	miR-2110	1.72	0.0406
	miR-6769a-5p	1.73	0.0022
	miR-7152-3p	1.77	0.0313
	miR-6750-5p	1.91	0.0040
	miR-200a-5p	2.10	0.0470
	miR-152-3p	2.60	0.0325
	miR-421	2.89	0.0005
	miR-149-5p	6.30	0.0022

n: number of individuals; DEmiR: differentially expressed miRNA; $p<0.05$.

Table S5. Cancer-specific and overall survival by clinicopathological features.

Survival	Clinicopathological Variables	p log-rank	HR (95% CI)	p
Cancer-specific survival	Lymph node metastasis (yes *vs* no)	0.003*	86.59 (0.18-41340.9)	0.156
	Perineural Invasion (yes *vs* no)	0.043*	4.71 (0.90-24.49)	0.066
	Lymphovascular Invasion (yes *vs* no)	0.240	2.40 (0.53-10.78)	0.255
	pT stage (T2+T3 *vs* T1)	0.183	28.64 (0.01-80176.9)	0.407
	Histological Grade (III *vs* I+II)	0.161	2.81 (0.62-12.68)	0.179
	HPV infection (presence *vs* absence)	0.276	0.33 (0.04-2.72)	0.301
	Tumor Size (> 5cm *vs* ≤ 5cm)	0.635	1.43 (0.32-6.41)	0.637
Overall survival	Lymph node metastasis (yes *vs* no)	0.062	3.45 (0.87-13.69)	0.078
	Perineural Invasion (yes *vs* no)	0.326	1.85 (0.53-6.45)	0.333
	Lymphovascular Invasion (yes *vs* no)	0.554	1.51 (0.38-5.90)	0.557
	pT stage (T2+T3 *vs* T1)	0.409	2.33 (0.30-18.45)	0.422
	Histological Grade (III *vs* I+II)	0.468	1.61 (0.44-5.83)	0.471
	HPV infection (presence *vs* absence)	0.349	0.48 (0.10-2.28)	0.359
	Tumor Size (> 5cm *vs* ≤ 5cm)	0.737	1.24 (0.35-4.42)	0.737

HR: Hazard Ratio; 95% CI: 95% Confidence Interval; * p <0.05.

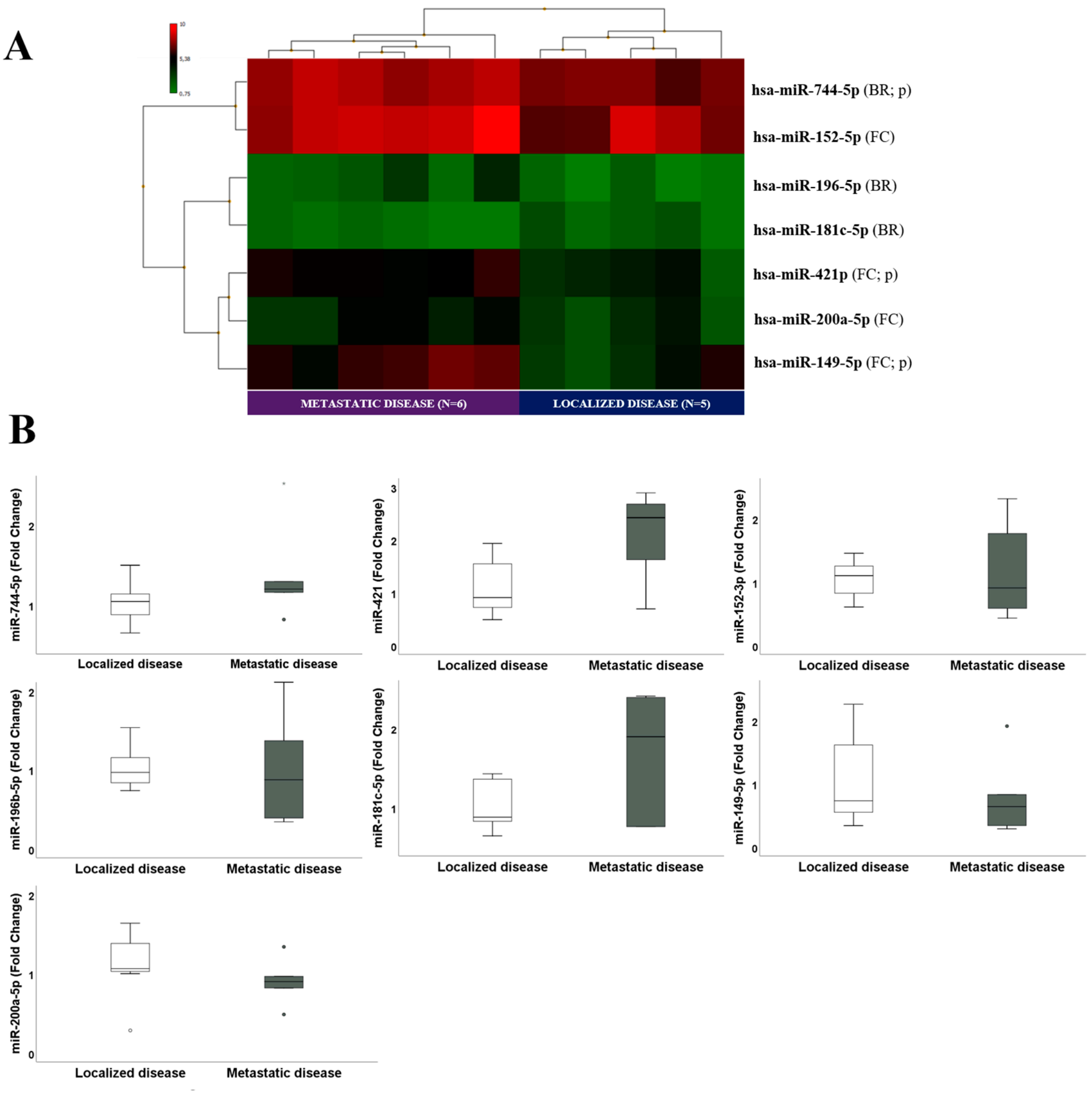

Figure S1. Differentially expressed miRNAs (DEmiRs) identified when comparing tumors of PeC patients with metastatic (*n*=6) in relation to localized disease (*n*=5) by microarray analysis and selected for validation in the independent cohort of PeC patients (*n*=13). (A) Hierarchical clustering analysis shows that the seven DEmiRs chosen for validation were able to cluster PeC samples according to lymph node metastasis. The reason for the selection of each DEmiR is indicated in parenthesis (FC: fold change; p: p-value and BR: Biological Relevance); (B) The boxplots show the comparison of the expression levels of the seven selected DEmiRs by qRT-PCR in tumors from patients with metastatic (*n*=6) in relation to localized disease (*n*=7) in the independent validation set of samples (*n*=13); PeC: penile cancer; qRT-PCR: quantitative Reverse Transcription Polymerase Chain Reaction.

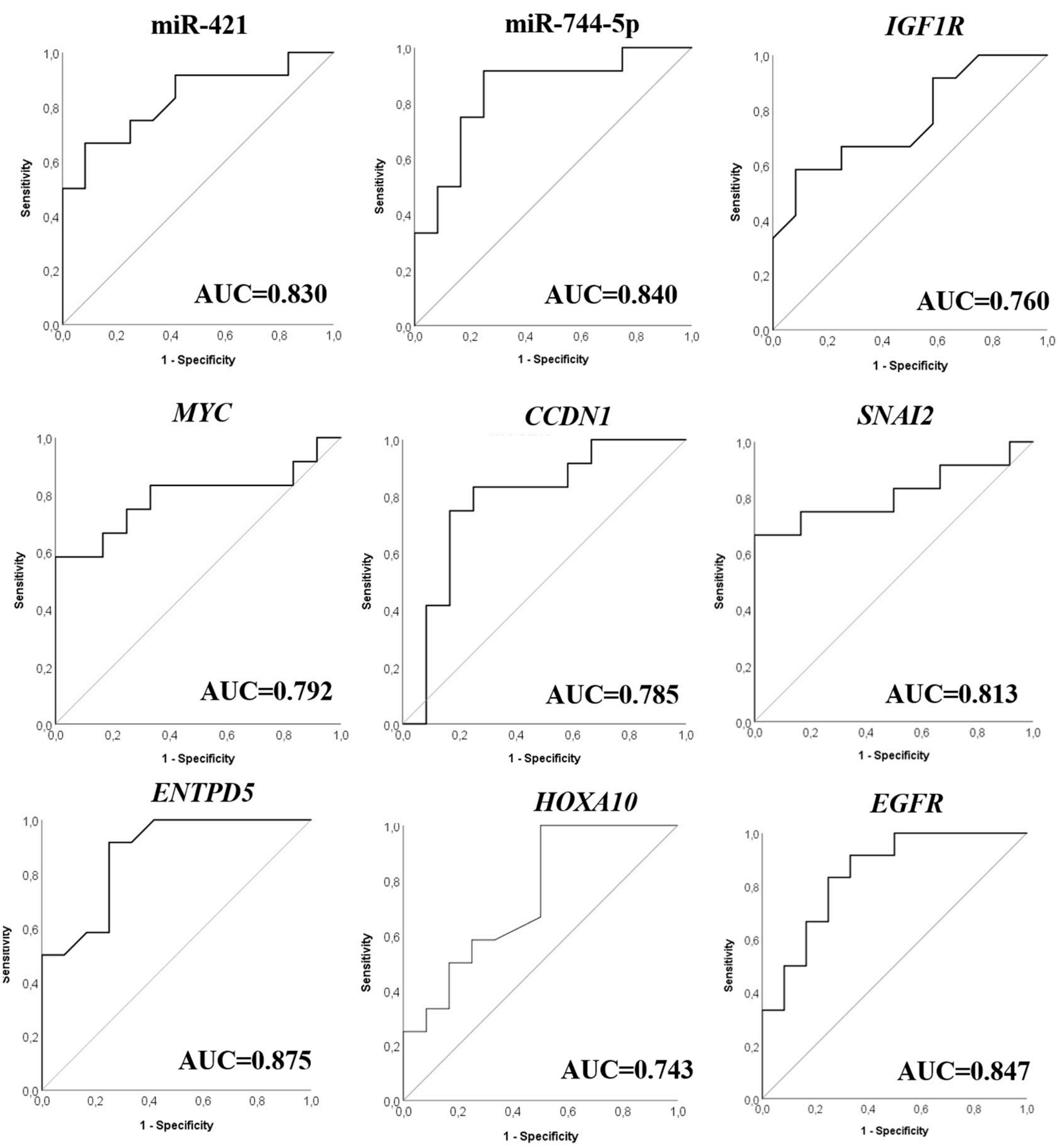

Figure S2. ROC curves of differentially expressed miRNAs (DEmiRs) and differentially expressed genes (DEGs) detected when comparing tumors of patients with metastatic and localized disease in the total sample of penile cancer patients (*n*=24), showing that they have good accuracy in differentiating these two groups; ROC: receiver operating characteristic; AUC: area under the curve.

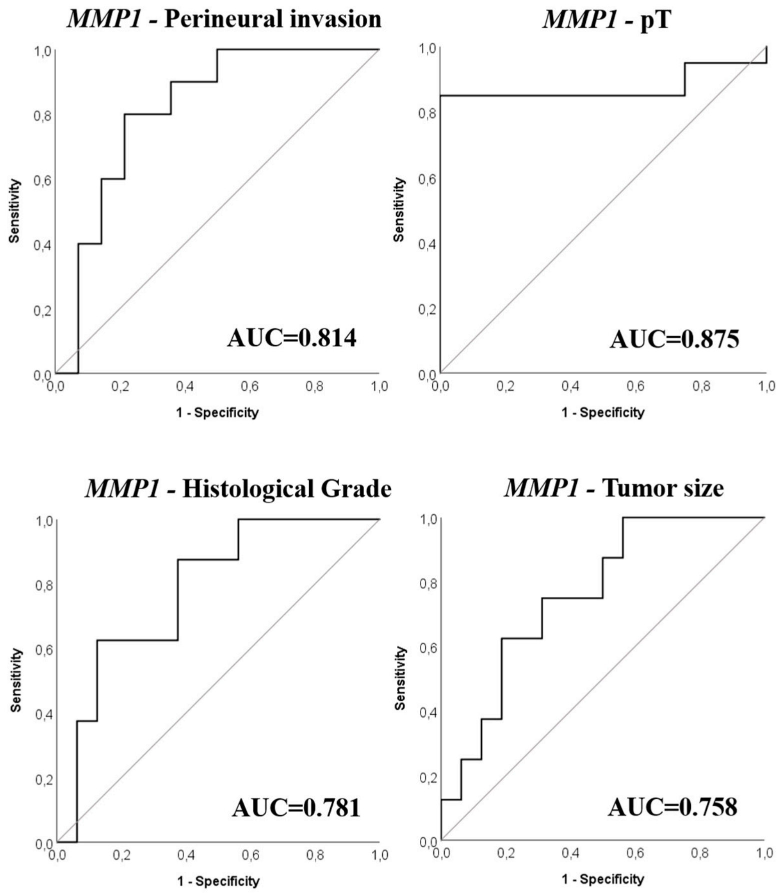

Figure S3. *MMP1* expression level is a good predictor of perineural invasion, worse pT stage (T2+T3), higher histological grade (III), and larger tumor size (> 5cm); AUC: area under the curve.

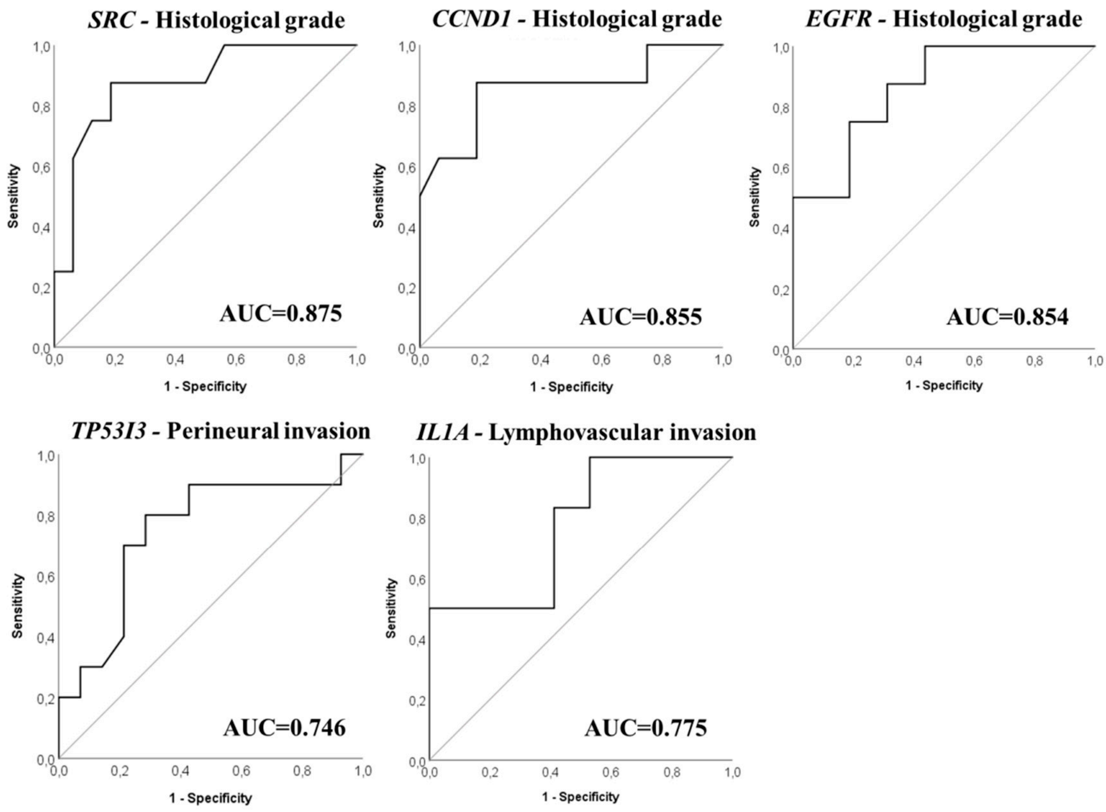

Figure S4. Receiver Operating Characteristic (ROC) curve analysis of mRNA expression levels and specific histopathological features; AUC: area under the curve.

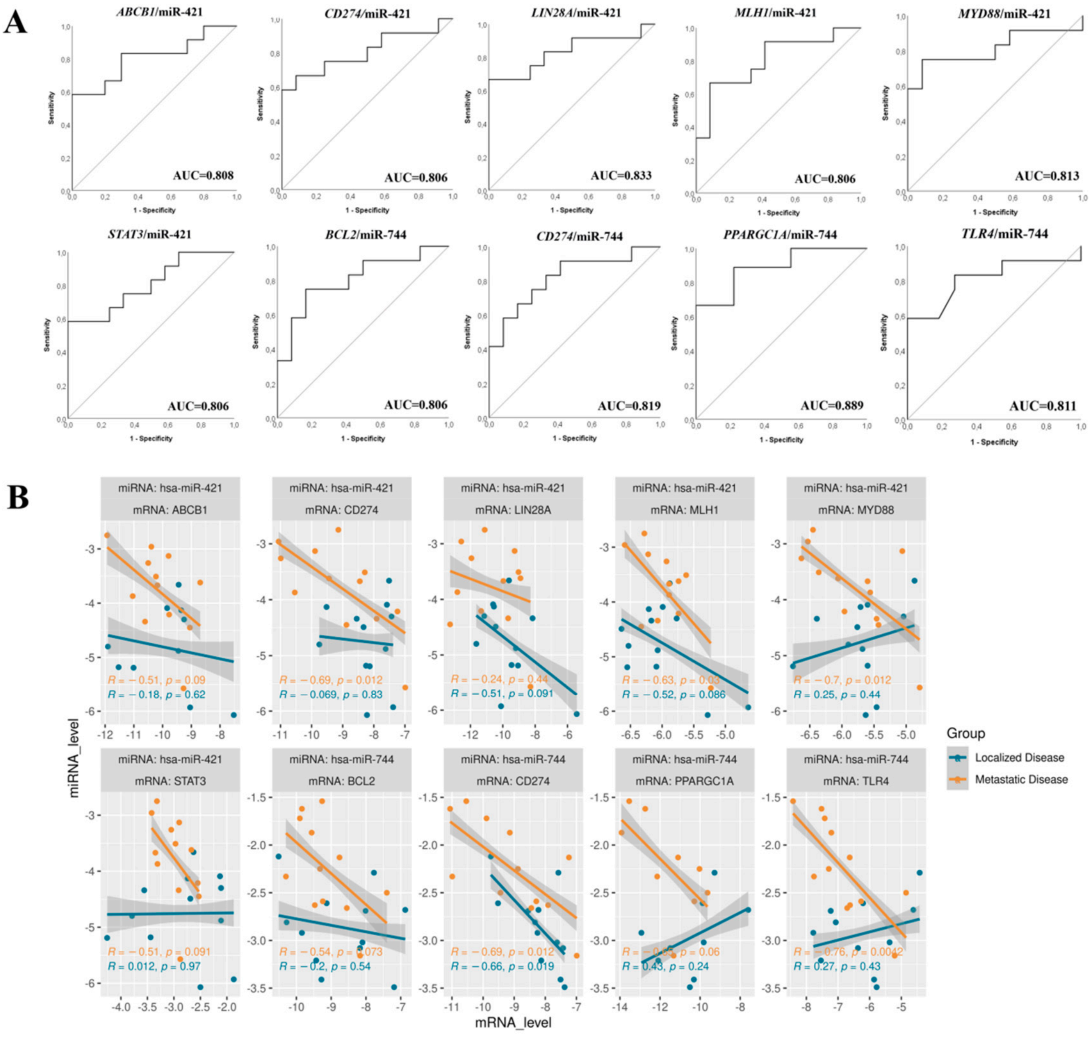

Figure S5. Ten mRNA-miRNA pair-wise ratios are proposed as candidates for prognosis biomarkers in Penile Cancer. (A) ROC curve analysis of mRNA-miRNA expression level ratios with good accuracy (AUC>0.80) for discriminating between tumors of patients with metastatic (n=12) and localized diseases (n=12). (B) mRNA and miRNA expression values of tumors of patients with metastatic and localized diseases were plotted for the ten miRNA-mRNA pairs. For each graph, the R coefficient and p values of the Pearson correlation test are indicated at the bottom. ROC: receiver operating characteristic; AUC: area under the curve.

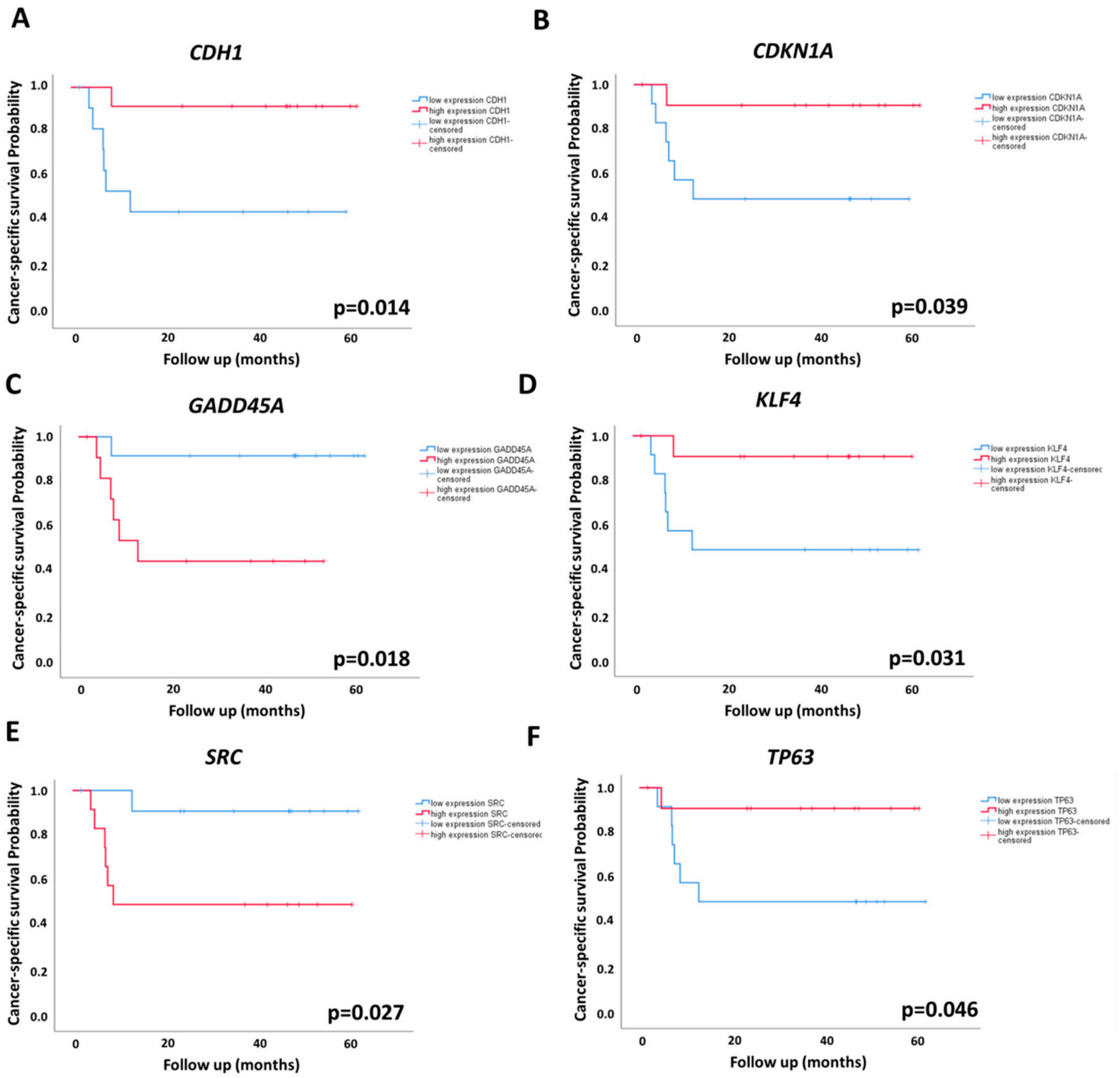

Figure S6. Cancer-specific survival (CSS) analyses of penile cancer patients according to gene expression patterns. Kaplan-Meier curves show that low expression (defined as values above the -dCT median expression) of *CDH1* (A), *CDKN1A* (B), *KLF4* (D), and *TP63* (F) and high expression levels of *GADD45A* (C) and *SRC* (E) were associated with shorter CSS.

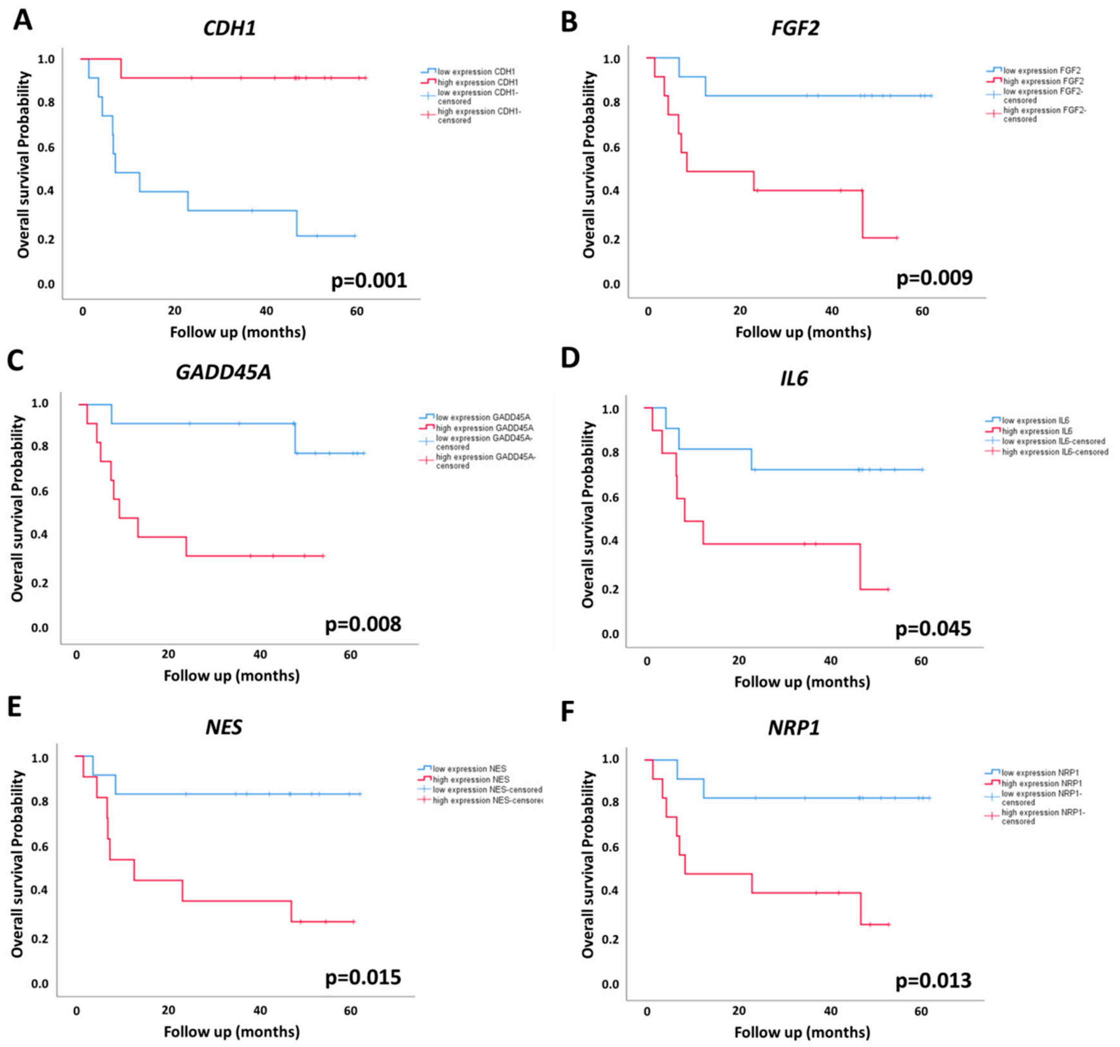

Figure S7. Overall survival (OS) analyses of penile cancer patients according to gene expression patterns. Kaplan-Meier curves show that low expression (defined as values above the -dCT median expression) of *CDH1* (A) and high expression levels of *FGF2* (B), *GADD45A* (C), *IL6* (D), *NES* (E), and *NRP1* (F) were associated with shorter OS.
